# Psychometric properties of the Chinese version of the preoperative assessment of readiness tool among surgical patients

**DOI:** 10.3389/fpsyg.2022.916554

**Published:** 2022-07-28

**Authors:** Guanjun Bao, Yuanfei Liu, Wei Zhang, Yile Yang, MeiQi Yao, Lin Zhu, Jingfen Jin

**Affiliations:** ^1^Quzhou College of Technology, Quzhou, China; ^2^The Second Affiliated Hospital Zhejiang University School of Medicine (SAHZU), Hangzhou, China; ^3^Women’s Hospital School of Medcine, Zhejiang University, Hangzhou, China; ^4^School of Nursing, Fudan University, Shanghai, China; ^5^Jinan People’s Hospital, Jinan, China; ^6^Changxing Branch Hospital of SAHZU, Huzhou, China; ^7^Key Laboratory of the Diagnosis and Treatment of Severe Trauma and Burn of Zhejiang Province, Hangzhou, China

**Keywords:** preoperative readiness, reliability, scale, surgical patients, validity

## Abstract

**Background:**

The evaluation of the surgical readiness of patients plays an important role in clinical care. Preoperative readiness assessment is needed to identify the inadequacy among surgical patients, which provides guide for interventions to improve patients’ preoperative readiness. However, there is a paucity of high-level, quality tool that evaluate surgical readiness of patients in China. The purpose of this study is to translate the Preoperative Assessment of Readiness Tool (PART) into Chinese and determine the reliability and validity of the Chinese version in the population of surgical patients.

**Methods:**

Using a standard translation-backward method, the original English version of PART was translated into Chinese. A convenient sampling of 210 surgical patients was recruited from 6 hospitals in Zhejiang Province to test the psychometric properties of this scale including internal consistency, split-half reliability, content validity, structure validity, and floor/ceiling effect.

**Results:**

A total of 194 patients (92%) completed questionnaires. The Chinese version of PART achieved Cronbach’s alphas 0.948 and McDonald’s omega coefficient 0.947, respectively, for the full scale. The estimated odd-even split-half reliability was 0.959. The scale-level content validity index was 0.867, and the items content validity index ranged from 0.83 to 1.0.The output of confirmatory factor analysis (CFA) revealed a two-factor model (χ^2^ = 510.96; df = 86; *p* < 0.001; root mean square error approximation = 0.08) with no floor/ceiling effect.

**Conclusion:**

The Chinese version of PART demonstrated acceptable reliability and validity among surgical patients. It can be used to evaluate patients’ preoperative preparation and help health professionals provide proper preoperative support.

## Background

With the development of medicine and technology, surgical services and treatments have seen incremental gains in patient outcomes. However, numerous postoperative complications such as infection, hematoma, and persistent postsurgical pain may pose a great threat to patients and compromise their quality of life (Kehlet, 2011). Studies have shown that sufficient preoperative preparation can reduce the incidence of surgical stress response and postoperative complications, and improve postoperative recovery (Carli, 2015; Azhar et al., 2016; Helander et al., 2019). Preoperative readiness refers to the patient’s evaluation of preoperative self-preparation. This concept emphasizes the active participation and cooperation of patients, family members and the medical team, which is beneficial to guarantee that patients meet the surgical requirements from both physical and psychological perspectives (Bolster and Manias, 2010). Previous research reported that medical staff paid more attention to the evaluation of patients’ physical and physiological preparation, such as physical examination and laboratory test, rather than a comprehensive evaluation of patients’ psychological and emotional preparation, information acquisition, as well as family and social support (Hines et al., 2010, 2015; Tho and Ang, 2016). Inadequate preoperative preparation may weaken the patient’s confidence in the surgery and lead to various negative consequences such as the delay of extubation time, the extension of postoperative recovery time, the increase of postoperative complications, and the reduction of postoperative satisfaction (Brubaker et al., 2014).

However, adequate preoperative preparation enables patients to undergo surgery in the best condition and guarantees surgical safety (Sawatzky et al., 2017). Martin et al. (2017) found that providing more surgery-related information was beneficial to improving patients’ preoperative preparation and reducing the readmission rate. Elkadry et al. (2003) showed that the evaluation of preoperative preparation of patients with cataracts had a positive correlation with the postoperative satisfaction of patients. A systematic review (Sau-Man Conny and Wan-Yim, 2016) also indicated that sufficient preoperative preparation could shorten hospital stay and reduce postoperative mortality. Therefore, the evaluation of preoperative readiness plays an important role in surgical patients’ preoperative preparation.

Although a considerable body of research has considered preoperative preparation, only a few studies have developed instruments for measuring preoperative readiness. Kenton et al. (2007) developed the preoperative readiness questionnaire including two dimensions of surgery-related knowledge and psychological stress, with a total of 11 items. It was mainly used for preoperative evaluation of female patients with urinary incontinence or pelvic organ prolapse in urology, obstetrics, and gynecology. Fakes et al. (2019) constructed a 27-item medical interventions preparation questionnaire, which was mainly used to evaluate the perceived self-preparation of patients during medical intervention (including medical imaging examination, radiotherapy and surgery). The colorectal cancer surgery questionnaire was developed by Carlsson et al. (2016) to evaluate the preoperative preparation of patients with colon cancer. However, these instruments only focused on a specific field and the application scope was limited. Moreover, most hospitals in China use preoperative readiness tools designed by the hospital-based on experience and lack reliability and validity evaluation. There is no reliable and valid Chinese version of patient readiness scale in China.

Torres and Macindo (2018) developed the preoperative assessment of Readiness Tool (PART) in 2018, which includes two factors: quality information acquisition and supportive interpersonal care assimilation, with a total of 15 items. This universal scale for the evaluation of patients’ preoperative readiness expanded the range of preoperative evaluation, which was likely to fill the gap in China. Therefore, we translated PART into Chinese and evaluate the psychometric properties of the Chinese version of this scale (including content validity, structural validity, internal consistency, split-half reliability, and floor/ceiling effect) in order to provide a scientific tool for the evaluation of preoperative preparation among surgical patients in China.

## Materials and methods

### Study design and participants

A cross-sectional, descriptive study was conducted in China from September 2021 to March 2022. A total of 210 patients from 6 hospitals in Zhejiang Province (including 4 upper tertiary hospitals, 1 middle tertiary hospital and 1 oncological hospital) were recruited according to the convenience sampling. The inclusion criteria were patients who (a) were above18 years old; (b) were ready for surgery within 24 h; (c) were able to give informed consent. The exclusion criteria were patients who (1) used antianxiety and antidepressant before operation; (b) were transferred to the emergency department due to changes in their health conditions; (c) were unconscious; (d) were unable to use a smart phone. We explained the purpose, benefits and risks of the study, and obtained informed consent from the patients. The investigation was conducted *via* online questionnaires which required an answer for every question so that there could not be any missing data. We guaranteed all the participants the confidentiality of their private information and their right to withdraw from the study at any stage.

### Instruments

#### Demographic characteristic

The self-developed demographic questionnaire was designed to collect the data of gender, age, educational level, religion, surgical experience, and department of patients.

#### Preoperative assessment of readiness tool

The English version of the PART was developed by Torres and Macindo (2018) in 2018 to evaluate the preoperative preparation of patients. The 15-item scale includes two domains: obtaining high-quality information and receiving supportive interpersonal care, which means preoperative patients feel ready for their operation by receiving quality medical information and incorporating supportive interpersonal care also aids surgical patients to feel ready. In PART, each item adopts a six-point Likert scale: 1 = “very disagree,” 2 = “disagree,” 3 = “a little disagree,” 4 = “a little agree,” 5 = “agree,” and 6 = “very agree.” The range of scores is from 15 to 90, with a high score indicating better preoperative preparation. Low [< 1 standard deviation (*SD*) below the mean], moderate (± 1 *SD* around the mean), and high (> 1 *SD* above the mean) levels of preoperative readiness were defined. The initial reliability of the PART was evaluated by internal consistency (Cronbach’s α = 0.97). The item level content validity index ranged from 0.67 to 1.00, and the scale level content validity index was 0.94.

#### Development of the Chinese version of the preoperative assessment of readiness tool

We had access to the original English version of the PART and obtained permission from the author to use and translate the scale into Chinese. Based on the guideline for the process of cross-cultural adaptation (Beaton et al., 2000), the translation stages were as followed:

Stage I: Translation: two nursing postgraduates who were proficient in English and Mandarin independently translated the scale into Chinese. After the integration of two researchers, one nursing professor with experience of studying aboard reviewed it with the two translators to form the first draft of the Chinese version of PART (PART-C).

Stage II: Back translation: two translators (one operating room nurse with a master’s degree and one graduate student majoring in English) who were completely blind to the original PART, translated PART-C back into English. After the integration of two translators, the back-translated version of PART was sent to the original author for clarification of words and sentences. Additionally, ambiguities and discrepancies were discussed by the five-member research group (two researchers, two translators and a professor of nursing). Finally, the second drafts of PART-C were formed. The integrated version, two back-translation versions, and translation issues were sent to the original author for confirmation.

Stage III: Cultural adaptation: Six experts (two surgical nursing experts, one operating room nursing expert, one senior nursing researcher, one nursing manager, and one nursing educationist) were invited to conduct a Delphi survey on the second draft of PART-C. The working experience of these experts was 15∼40 (28.50 ± 7.50) years. A 4-level Likert scale was adopted to assess content equivalence (1 = not relevant, 2 = unable to assess relevance, 3 = relevant but needs minor alteration, 4 = very relevant and succinct), including whether the descriptions of the items were clear, whether the item contents were relevant and whether the items were adapted to Chinese cultural background (Tang and Dixon, 2002). A consensus was achieved through the discussion for revision among five-member research group, the third draft of PART-C was developed.

Stage IV: Pilot study: 30 patients who met the eligibility requirement in a tertiary hospital in Zhejiang province were selected for the pilot study (Beaton et al., 2000). Before the questionnaire survey, we informed the patients of the purpose and significance of the study. Patients were encouraged to express whether they understood each item and how they feel about the scale in their own words. In this stage, patients showed agreement that there were no difficulties in responding to all items. The time required for the answer was recorded.

**FIGURE 1:**
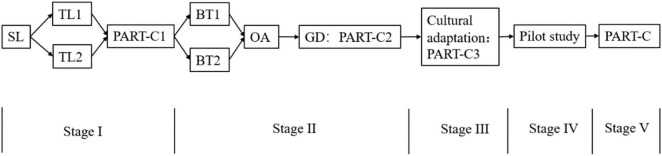
The structured flowchart of the translation process. SL, Source Language; TL, Target Language; BT, Back Translation; OA, Original Author; GD, Group Discussion.

Stage V: Based on the patients’ responses, expert panel comments, and research group’s feedback, the final PART-C was generated to evaluate psychometric properties. The original PART and PART-C were attached in the Supplementary Materials. A structured flowchart of the translation process is presented in Figure 1.

### Data collection

Six nurses were trained for collecting the data through online questionnaires. The survey consisted of two parts, the demographic form to collect participants’ characteristics, and the PART-C. Investigators explained the purpose, benefits and risks of the research in a simple and unified language to the patients, and asked them to sign the informed consent. The patients scanned the QR code through WeChat app to complete the questionnaires. We deleted the invalid questionnaires according to the following standards: (1) the finish time was within 1 min (typically it took ∼ 5 min to complete the scale) (Huang et al., 2012); (2) all answers were the same, including general information.

### Data analysis

All data analyses were performed using SPSS version 25.0, AMOS version 24.0, and jamovi version 2.2.5. Two-tailed tests were calculated with a *p*-value of 0.05 as the significance level. Descriptive analyses were used to report the mean and standard deviation (*SD*) of the continuous variables and percentage frequency for the categorical variables. We tested the normality of the distribution of continuous variables using the Kolmogorov-Smirnov test. Since the scores of PART-C were normally distributed, the difference testing between groups was performed using Student’s *t*-test or one-way analysis of variance (ANOVA).

### Content validity

Content validity describes the degree to which the content of an instrument adequately reflects the construct to be measured (Souza et al., 2017). Experts’ ratings on the assessment of content validity indexes at the item level (I-CVI) and the scale level (S-CVI) were calculated, using a 4-level Likert scale: 1 = irrelevant, 2 = weak correlation, 3 = strong correlation, and 4 = very correlation (Polit et al., 2007). The I-CVI was computed as the number of experts who gave a rating of 3 or 4 to the relevancy of each item divided by the total number of experts. The S-CVI was calculated as the average of the content validity of all items.

### Structure validity

Structure validity indicates whether the scores of an instrument adequately reflect the dimensionality of the construct to be measured. Maximum likelihood confirmatory factor analysis (CFA) was conducted to evaluate the structural validity of the PART-C. Our sample size in this study met the requirement of recommendation for CFA (100–400 is deemed adequate) (Hair et al., 2010). A non-significant chi-square Index (χ^2^) is desirable. Standardized root means square residual (SRMR) and root mean square error of approximation (RMSEA) are also suitable to assess the goodness of fit. Therefore we relied on the following standards to evaluate model fit: SRMR ≤ 0.08, RMSEA ≤ 0.08, goodness-of-fit index (GFI) > 0.90, comparative fit index (CFI) > 0.90 (Kline, 2005). Also, GFI and CFI above 0.85 and RMSEA below 0.10 were judged to be acceptable as marginal fit (Browne and Cudeck, 1993). Besides, modification indices (MIs) were inspected to improve the fit of the model.

### Reliability

Reliability refers to the consistency and stability of the results measured by the scale. The reliability test adopts internal consistency reliability (Cronbach’s α Coefficient and McDonald’s omega coefficient) and split-half reliability (Souza et al., 2017). The items were divided into two halves by odd and even. The correlation coefficient of the scores of the two halves was calculated. Internal consistency reliability and split-half reliability of 0.80 or higher were considered acceptable (McNeish, 2018).

### Floor/ceiling effect

The floor/ceiling effect occurs when there is some lower/upper limit on a questionnaire and a large percentage of respondents score near this limit, which may compromise the accuracy of study results. The floor effects for the scale were determined by the percentage of the sample size that got the lowest score, and the ceiling effects were assessed by the percentage of the respondents that achieved the highest score. Less than 15% of patients achieving the highest or lowest score in the PART-C were deemed as no floor and ceiling effects (Terwee et al., 2007).

### Ethical consideration

This study was approved by the Ethics Committee of the Second Affiliated Hospital Zhejiang University School of Medicine (SAHZU, number: 2021–0727). The ethical principles of voluntary participation, anonymity, and confidentiality were guaranteed. All the participants voluntarily participated and signed informed consent. This study was conducted according to the Declaration of Helsinki.

## Results

### Translation and cultural adaptation

The bilingual translators accurately translated the PART into Chinese, which was confirmed by the five-member research group. Additionally, the original author endorsed the back-translated English version as retaining the original meaning. However, due to the differences in cultural background and current status in hospitals in China, there were some revisions achieved by the discussion of research group: item 1 “I am ready because I prayed for strength.” was revised as “I am ready because I wished for strength” because of non-religious background in most of the Chinese, and item 10 “I am ready because I checked my physician’s track record.” was revised as “I am ready because I understood my physician’s treatment and medication.” because the patients in China rarely had access to physician’s track record. Further information is presented in Tables 1, 2.

**TABLE 1:** Further information for the translation process.

Stage	Result	Problem	Example	Solution
I	First draft of PART-C	Discrepancies were related to the wording or phrasing of the items which were synonyms in Chinese. There was no difference between sentence structures.	Item 3: “comforting” was translated to “安心” vs. ”安慰”. Item 8: “concern” was translated to “担忧” vs. “忧虑”.	Two translators discussed with one nursing professor to decide the proper wording and phrasing.
II	Second draft of PART-C	Discrepancies were caused by different expressions or synonyms when comparing the two back translations to the original English version.	Item 4: “confirmed my understanding of the operation” in the back translation was corresponding to “confirmed what I know about my operation” in the original version. Item 3, 4, 8: “medical staff” in the back translation was a synonym for “healthcare providers” in the original version.	Original author was consulted for confirmation and endorsed that the back translation retained the original meaning. Therefore, translations did not require rewording.
III	Third draft of PART-C	Two items were found inappropriate in the context of culture and clinical setting in China.	Item 1: “I am ready because I prayed for strength.” was incompatible with the non-religious context. Item 10: “I am ready because I checked my physician’s track record.” was inappropriate in clinical scenarios in China.	An expert panel consisting of six experts was consulted.
IV	/	Some of the participants needed the help to read aloud the content of the scale due to the poor eyesight. There were no difficulties in responding to all items.	/	The researchers read aloud the items without any leading information.
V	Final PART-C	A few of the participants rated the same answers sequentially, which reminded the researchers to avoid invalid responses in the survey.	/	The researchers were trained to collect the data, e.g., choosing the most convenient time for the participants and using some incentives.

**TABLE 2:** Modifications of items and reasons.

Item	Before	After	Reason
Item 1	I am ready because I prayed for strength.	I am ready because I wished for strength.	Most of the Chinese people have non-religious background. The wording has been changed to enlarge the applied situation.
Item 10	I am ready because I checked my physician’s track record.	I am ready because I understood my physician’s treatment and medication.	The patients in China rarely have access to physician’s track record. Typically, the physician tells them the information about their treatment and medication.

### Participant characteristics

Of the 210 patients, 194 completed questionnaires for analysis, with a response rate of 92.38%. Most of the patients were 31–59 years (55.33%), female (53.33%), non-religious (87.11%), had no surgical experience (55.56%), and had a bachelor degree or above (40%). They are recruited from different departments: breast surgery, hepatobiliary surgery, gynecology surgery, gastrointestinal surgery, orthopedics surgery, thoracic surgery, and other departments. Further details are presented in Table 3.

**TABLE 3:** The characteristics of the participants (*N* = 194).

Characteristic	Frequency (*f*)	%
**Age (years)**		
18–30	24	12.37%
31–59	107	55.33%
≥60	63	32.30%
**Sex**		
Male	91	46.67%
Female	103	53.33%
**Educational level**		
Primary school	69	35.56%
Secondary or high school	30	15.56%
College	17	8.89%
Bachelor or above	78	40%
**Religious background**		
No	169	87.11%
Yes	25	12.89%
Previous surgical experience		
No	108	55.56%
Yes	86	44.44%
**Department**		
Breast surgery	26	13.33%
Hepatobiliary surgery	32	16.67%
Gynecology surgery	26	13.33%
Gastrointestinal surgery	29	14.95%
Orthopedics surgery	20	10.31%
Thoracic surgery	27	13.92%
Other	34	17.52%

### Content validity

Using six experts and an averaging calculation method, the I-CVIs of the total 15 items were from 0.83 to 1.0. The S-CVI was 0.867. According to experts’ views, the PART-C reflected the framework of preoperative preparation and had logical consistency with the English version. Moreover, the PART-C was understandable and acceptable for measuring preoperative readiness among the Chinese surgical patients.

### Structural validity

Data indicated that the PART-C items were suitable for factor analysis (Kaiser-Meyer-Olkin index = 0.913 and Bartlett’s test of sphericity *p* < 0.001) and demonstrated a two-factor structure. The results of CFA were consistent with the original findings, however, the initial model indices suggested a poor fit. The MIs showed potential misfits within the questionnaire, as the two items that asked about supportive interpersonal care were related to each other (item 12 “I am ready because I listened to and followed instructions from my doctor and nurses.” and item 13 “I am ready because I am receiving professional care and treatment”). By examining the model fit statistics through pairing items 12 and 13, it demonstrated an improvement in all the indices and achieved a better acceptable fit [χ^2^ = 510.963, df = 86 (*p* < 0.05), SRMR = 0.08, RMSEA = 0.08, GFI = 0.867, CFI = 0.849], indicating an acceptable fit. The CFA model for PART-C is shown in Supplementary Figure 1.

### Reliability test

The results showed that Cronbach’s α coefficient of the PART-C was 0.948, with subscales achieving 0.922 and 0.896, respectively (Table 4). The McDonald’s omega coefficient was 0.947 for total and the odd-even split-half reliability of the scale was 0.959. These findings indicated that the internal consistency of the PART-C was excellent.

**TABLE 4:** The reliability of the PART-C.

Subscale	Cronbach’s α coefficient	ICC [95% confidence interval]	McDonald’s omega coefficient	ICC [95% confidence interval]
Quality information acquisition	0.922	0.887, 0.947	0.919	0.876, 0.946
Supportive interpersonal care assimilation	0.896	0.847, 0.932	0.895	0.838, 0.934
Total scale	0.948	0.923, 0.965	0.947	0.917, 0.966

### Floor/ceiling effect

No participant (0%) achieved the lowest possible score (15) and the highest (90), demonstrating no floor or ceiling effect was detected.

### Preoperative readiness score

The average score of patients’ preoperative readiness was 77.37 ± 10.26 (ranging from 15 to 90). Low, medium and high level of preoperative readiness were regarded as < 1 *SD* below the mean, ± 1 *SD* around the mean, and > 1 *SD* above the mean, which were < 67.11, 67.11–87.63 and > 87.63, respectively. 84.02% of surgical patients had a low to medium level of preoperative readiness, and among them, 9.28% had a low level of preoperative readiness. The *t*-test and ANOVA showed that there was no statistically significant difference in PART-C scores among patients with different clinical departments and previous surgical experience.

## Discussion

In this study, the English version of the PART was translated into Chinese according to the methodology guideline (Beaton et al., 2000), which provides profound guidance for cross-cultural translation. Meanwhile, the equivalence of concept, semantics, and content was realized. We strictly followed the principles of operational equivalence and standard equivalence in the study process. The Chinese version PART had acceptable reliability and validity in this study, with no floor/ceiling effect. A total of 15 items in the scale made it simple and easy to understand and could be completed in 5 min. Due to its good feasibility and acceptability, PART-C is the appropriate tool to assess the preoperative readiness of surgical patients in China.

In the cultural adaptation stage, two items were modified based on the cultural differences. Contrary to the Christian religion in western countries, Chinese culture was deeply influenced by Confucianism, Taoism and Buddhism. Since the Cultural Revolution in the history, a large number of people in mainland China became atheist (Wu and Wu, 2012). The religious practices in western countries were not suitable in Chinese culture. Therefore, we changed the term “pray” to “wish” in item 1, which was similar to the modification and adaptation of spiritual care in previous study (Xie et al., 2019). In addition, the asymmetry of medical information between physicians and patients was one of the major issues in China, which made patients vulnerable in clinical decisions (Lyu et al., 2016). It was less likely for the patients to checked physician’s track record, thus, we modified the item to fit the situation in China.

Preoperative readiness of patients is a dynamic process, which involves the joint participation of patients, their families and medical staff to make preparation for patients from physiological, psychological, and social perspectives (Carlsson et al., 2016). Studies showed that sufficient preoperative preparation would enhance patients’ confidence in the operation, and enable patients to maintain a positive mindset, thus, bringing numerous benefits to the postoperative recovery (Sawatzky et al., 2017). Therefore, it is very necessary for health professionals to assess patients’ preoperative readiness before operation. PART-C is validated to be an ideal tool for evaluation. The scale includes two dimensions: quality information acquisition (9 items) and supportive interpersonal care assimilation (6 items). As for the content of the items, it involves physical domain such as “I think I’m ready because I understand the risks of surgery” (e.g., complications, disability, physical changes, etc.), psychological domain such as “I am ready because my healthcare providers are comforting,” and social support domain such as “I think I’m ready because I know my family will support and take care of me.” Even though the assessment of preoperative preparation has attracted more and more attention, there is a dearth of a universal scale of preoperative readiness in China. Therefore, the translation and validation of PART in Chinese population lay a solid foundation for future research related to preoperative preparation.

The results of this study showed that the average score of patients’ preoperative readiness was 77.37 ± 10.26 (ranging from 15 to 90), which was slightly lower than that of Torres and Macindo (2018) (80.68 ± 14.89). Low, medium, and high preoperative readiness were < 67.11, 67.11-87.63 and > 87.63, respectively. In this study, 84.02% of surgical patients had a low to medium level of preoperative readiness, and among them, 9.28% had a low level of preoperative readiness, which helped spot the target patients. The variation in preoperative readiness may be related to the severity of the disease. Some patients with malignant cancer who will undergo a major surgery may have more concern and a lower level of preoperative readiness while some patients prior to a minor surgery are more likely to have a higher level of preoperative readiness (Rockefeller et al., 2021). Therefore, additional attention should be paid to the patients with lower preoperative readiness to explore the reasons for their poor readiness and give a detailed guide to improve their preoperative readiness.

Reliability reflects the consistency and stability of the scale, which was evaluated by Cronbach’s α coefficient, McDonald’s omega coefficient and split half reliability in our study. The Cronbach’s α coefficient and McDonald’s omega coefficient of this PART-C were 0.948 and 0.947, respectively, indicating that the scale has excellent reliability. Content validity reflects the extent to which the instrument actually measures the target construct (Polit et al., 2007). One of the key points to ensure the effective measurement of content validity is the authority of experts in the surgical field (Terwee et al., 2018). The six experts in this study had rich clinical experience and a high level of position. The expert authority indexes were 0.753 ∼ 0.957. The scale content validity index of the scale was 0.867, and item content validity indexes were 0.830 ∼ 1.000, demonstrating sound content validity. The CFA of this study showed a two-dimension structure of the scale, with χ^2^/DF = 5.941, RME = 0.08, GFI = 0.867, CFI = 0.85, which met the requirements for acceptable structural validity (Brown, 2006; Schober et al., 2021). The test-retest reliability was not able to be evaluated due to the short period of time before operation. Altogether, the psychometric properties of PART-C were acceptable, which can be used to evaluate the preoperative readiness of patients in China.

There are several potential clinical implications for future research and clinical practice. First of all, this scale can help health professionals evaluate the preoperative readiness of surgical patients, and explore the factors affecting their preoperative readiness, which may be beneficial to them to carry out strategies to improve patients’ preoperative readiness. These probably reduce surgical stress and the incidence of postoperative complications, and improve patients’ satisfaction. Secondly, the introduction of this instrument paves the way for the research related to preoperative preparation. The factor structure in the study may be affected by the norms of the sample and social culture. More research needs to be done to make an appropriate adaptation of this tool based on Chinese cultural background and retest it in a larger population sample. The verification of PART-C in different cultural backgrounds will enhance the universality of the scale.

To the best of our knowledge, this is the first study in China to introduce an instrument regarding preoperative readiness. We translated the PART into Chinese according to the Beaton guideline which considers cross-cultural adaptation to produce content equivalency between source and target language. This would be helpful to ensure retention of psychometric properties at an item and a scale level. However, there are also some limitations in the study. To begin with, compared with other recent guidelines, we did not conduct the preliminary psychometric test among a bilingual population (Sousa and Rojjanasrirat, 2011). This process helps support the content and construct validity of the instrument prior to full psychometric evaluation but is rarely used due to the inaccessibility of bilingual individuals. Beside, the validation of the translation was not sufficient in our study. The original version and back-translated version are recommended to be compared in terms of comparability of language and similarity of interpretability according to Sperber (2004) guideline. Second, according to the rules of thumb related to sample size for CFA, two to three times the amount of 10 participants per variable was recommended (Terwee et al., 2007). Larger sample size is required for further research. Another limitation was that the participants of this study were mainly from Zhejiang Province, China, which had the potential of regional limitations and the samples were not sufficiently representative. Therefore, it is recommended to recruit participants from more religions and different levels of hospitals to verify the reliability and validity of the scale. Moreover, due to the limited resources, we failed to reach out the English language native speakers at the back translation stage. However, in order to guarantee the quality of back translation, we invited two bilingual translators who were proficient in English and blinded to the original English version of the scale. Both of them had study abroad experience. The original author also endorsed the back-translated English version as retaining the original meaning. Finally, to explore the possible reasons for different preoperative readiness, we have conducted relevant statistical analysis such as *t*-test and ANOVA. However, we did not find statistically significant differences of the PART-C scores among patients with different clinical departments and previous surgical experience. Future research should take more variables into consideration to evaluate the factors that influence the preoperative preparation of patients.

## Conclusion

The findings of this cross-sectional study identified the Chinese version of PART as a reliable and valid scale with good utility. The PART-C can be a practical tool to evaluate the preoperative readiness of surgical patients in China. Health professionals can use it to help patients enhance their preoperative preparation for perioperative management. Further empirical evidence supporting its application is expected from the ongoing assessment of the PART-C in Chinese population.

## Data availability statement

The raw data supporting the conclusions of this article will be made available by the authors, with reasonable request.

## Ethics statement

The studies involving human participants were reviewed and approved by the Second Affiliated Hospital Zhejiang University School of Medicine, Hangzhou, China. The patients/participants provided their written informed consent to participate in this study.

## Author contributions

GB designed and conducted the study. YL and GB were major contributors in drafting the manuscript. WZ, YY, and LZ helped with data collection. MY and JJ made substantial inputs to the revision of the article. All authors approved the final manuscript.

## Conflict of interest

The authors declare that the research was conducted in the absence of any commercial or financial relationships that could be construed as a potential conflict of interest.

## Publisher’s note

All claims expressed in this article are solely those of the authors and do not necessarily represent those of their affiliated organizations, or those of the publisher, the editors and the reviewers. Any product that may be evaluated in this article, or claim that may be made by its manufacturer, is not guaranteed or endorsed by the publisher.

## Abbreviations

PART: Preoperative Assessment of Readiness Tool
PART-C: Chinese version of Preoperative Assessment of Readiness Tool
*SD*: standard deviation
CFA: Confirmatory Factor Analysis models
SRMR: Standardized root mean square residual
RMSEA: Root mean square error of approximation
CFI: Comparative fit index.
GFI: Goodness-of-fit index
