# Supplementary material for: Psychometric properties of the Chinese version of the preoperative assessment of readiness tool among surgical patients
Source: Front Psychol. 2022 Jul 28;13:916554. doi: 10.3389/fpsyg.2022.916554 (PMC9366670; doi:10.3389/fpsyg.2022.916554)
Supplement: Supplementary file 1 [file Data_Sheet_1.docx]

Item9

Item8

Item7

Item5

Item6

Item3

Item4

Item1

Item2

Item10

Item11

Item12

Item13

Item14

Item15

Fig. 1. CFA Model for the PART-C
